# Kisspeptin treatment improves fetal-placental development and blocks placental oxidative damage caused by maternal hypothyroidism in an experimental rat model

**DOI:** 10.3389/fendo.2022.908240

**Published:** 2022-07-28

**Authors:** Bianca Reis Santos, Jeane Martinha dos Anjos Cordeiro, Luciano Cardoso Santos, Erikles Macedo Barbosa, Letícia Dias Mendonça, Emilly Oliveira Santos, Isabella Oliveira de Macedo, Mário Sergio Lima de Lavor, Raphael Escorsim Szawka, Rogeria Serakides, Juneo Freitas Silva

**Affiliations:** ^1^ Centro de Microscopia Eletronica, Departamento de Ciencias Biologicas, Universidade Estadual de Santa Cruz, Campus Soane Nazare de Andrade, Ilheus, Brazil; ^2^ Departamento de Fisiologia e Biofísica, Instituto de Ciencias Biologicas, Universidade Federal de Minas Gerais, Belo Horizonte, Brazil; ^3^ Departamento de Clinica e Cirurgia Veterinarias, Escola de Veterinaria, Universidade Federal de Minas Gerais, Belo Horizonte, Brazil

**Keywords:** kisspeptin, trophoblast, thyroid, cell stress, inflammation, fetal development, rat

## Abstract

Maternal hypothyroidism is associated with fetal growth restriction, placental dysfunction, and reduced kisspeptin/Kiss1R at the maternal-fetal interface. Kisspeptin affects trophoblastic migration and has antioxidant and immunomodulatory activities. This study aimed to evaluate the therapeutic potential of kisspeptin in the fetal-placental dysfunction of hypothyroid Wistar rats. Hypothyroidism was induced by daily administration of propylthiouracil. Kisspeptin-10 (Kp-10) treatment was performed every other day or daily beginning on day 8 of gestation. Feto-placental development, placental histomorphometry, and expression levels of growth factors (VEGF, PLGF, IGF1, IGF2, and GLUT1), hormonal (Dio2) and inflammatory mediators (TNFα, IL10, and IL6), markers of hypoxia (HIF1α) and oxidative damage (8-OHdG), antioxidant enzymes (SOD1, Cat, and GPx1), and endoplasmic reticulum stress mediators (ATF4, GRP78, and CHOP) were evaluated on day 18 of gestation. Daily treatment with Kp-10 increased free T3 and T4 levels and improved fetal weight. Both treatments reestablished the glycogen cell population in the junctional zone. Daily treatment with Kp-10 increased the gene expression levels of *Plgf*, *Igf1*, and *Glut1* in the placenta of hypothyroid animals, in addition to blocking the increase in 8-OHdG and increasing protein and/or mRNA expression levels of SOD1, Cat, and GPx1. Daily treatment with Kp-10 did not alter the higher protein expression levels of VEGF, HIF1α, IL10, GRP78, and CHOP caused by hypothyroidism in the junctional zone compared to control, nor the lower expression of *Dio2* caused by hypothyroidism. However, in the labyrinth zone, this treatment restored the expression of VEGF and IL10 and reduced the GRP78 and CHOP immunostaining. These findings demonstrate that daily treatment with Kp-10 improves fetal development and placental morphology in hypothyroid rats, blocks placental oxidative damage, and increases the expression of growth factors and antioxidant enzymes in the placenta.

## Introduction

Kisspeptin, encoded by the gene *Kiss1*, was first purified from human placenta (1, 2). Soon after kisspeptin was discovered, it was recognized as essential for fertility, as it regulates gonadotropin-releasing hormone (GnRH) secretion by the hypothalamus through the G protein-coupled receptor GPR54 (Kiss1R). Therefore, failures in the kisspeptin/Kiss1R signaling system result in hypogonadotropic hypogonadism (3, 4). In addition to its hypothalamic action, however, the kisspeptin/Kiss1R system is expressed in a variety of tissues, including the placenta (5, 6), although studies on the action of kisspeptin on the placenta are still scarce (7–9).

In humans, circulating levels of kisspeptin are low in most physiological conditions, except in the final third of gestation, when these levels increase almost 10, 000 times, after which they quickly return to basal level after delivery (10–12). This suggests that the placenta is one of the main sources of systemic kisspeptin. Studies have shown that kisspeptin influences the adhesion and implantation of blastocysts and the decidualization and migration of trophoblasts, and regulates the immunological profile and uterine natural killer cells (uNKs) (5, 7–9, 11–17), suggesting that faults in its expression in the maternal-fetal interface may be involved in placental disorders (12, 18).

Gestational diseases such as preeclampsia, gestational hypertension, miscarriage, gestational diabetes, and obesity have altered plasma and/or placental levels of kisspeptin (17–30). Therefore, its plasma profile can be assessed to predict gestational success (12, 18). We have recently demonstrated that rats with maternal hypothyroidism, another important gestational disease, also exhibit decidual and placental reduction of the kisspeptin/Kiss1R system (31).

Women with maternal hypothyroidism are more likely to suffer miscarriage and intrauterine growth restriction (Silva et al., 2012; Silva, Ocarino, and Serakides, 2018). Moreover, plasma levels of triiodothyronine (T3) and thyroxine (T4) are low in patients with preeclampsia (32, 33). Studies in rats have also shown that hypothyroidism reduces intrauterine trophoblast migration and proliferation, increases placental apoptosis, compromises placental morphogenesis and vascularization, causes oxidative and reticular stress, and alters the immune profile and uNK cell population at the maternal-fetal interface (34–40). However, the role of kisspeptin in these placental changes is still unknown.

Although T4 replacement is the first choice in patients with hypothyroidism, some patients are refractory to T4 replacement (41) and need other therapeutic alternatives when they need to treat a medical condition, such as infertility problems. In this sense, exogenous kisspeptin has been shown to restore ovarian function in hypothyroid rats (42). Furthermore, studies have shown that kisspeptin administration has an immunomodulatory effect on gestation (15) and an antioxidative effect on the ovary (43), liver (44), and testicle (45), and blocks the occurrence of reticular stress in hypothalamic GT1-7 cell line (46). Therefore, this study aimed to evaluate the therapeutic potential of kisspeptin in placental dysfunction caused by maternal hypothyroidism. We demonstrate that daily kisspeptin treatment improves development of the fetus and placenta in hypothyroid rats, increases the expression of growth factors and antioxidant enzymes by the placenta, and positively modulates oxidative stress, reticular stress and immune mediators, thus characterizing the therapeutic potential of kisspeptin in a gestational disease for the first time.

## Materials and methods

### Animal management and induced hypothyroidism

Adult Wistar rats (200-250 grams) were kept in plastic boxes with controlled temperature (22 ± 2°C) and brightness (12 h light/12 h dark), and water and feed *ad libitum*. The animals were equally distributed into euthyroid (control) (n=13), hypothyroid (n=15), and hypothyroid groups treated with kisspeptin-10 (Kp10) every other day (KpT1; n=15) or every day (KpT2; n=15). Hypothyroidism was induced by administering 6-propyl-2-thiouracil (PTU) (4 mg/Kg/day) through an orogastric tube every day, starting five days before mating, while the control group received water as a placebo (35, 36). All experimental procedures were approved by the Ethics Committee on the Use of Animals of Santa Cruz State University (UESC) (Protocol 036/16).

Five days after the start of treatment with PTU, five animals from each group were euthanized by decapitation for blood collection and dosage of free T4 to confirm the induction of hypothyroidism (Control, 1.20 ± 0.05 µg/dL; Hypothyroid, 0.47 ± 0.09 µg/dL; KpT1, 0.52 ± 0.06 µg/dL; KpT2, 0,42 ± 0.08 µg/dL (P<0.01)). Vaginal cytology was performed in the remaining rats, and the animals in proestrus were housed with fertile adult males for mating. The presence of spermatozoa in the vaginal cytology the next morning confirmed copulation and was defined as day 0 of gestation (0 GD). All animals from control group were mated and became pregnant, while from hypothyroid groups (Hypothyoid; KpT1; KpT2) around 86.6% of the females became pregnant.

### Treatment with kisspeptin-10

The hypothyroid animals treated with Kp10 were distributed into two groups, one treated every other day (KpT1) and one treated daily with Kp10 (KpT2). Treatment was initiated on the 8th GD (8 µg/Kg/day) (Cat. No. 4243, Tocris Bioscience, Bristol, UK), intraperitoneally, and was maintained until the day of euthanasia. Kp10 treatment was initiated on the 8th GD so that it would not influence embryo implantation. The animals in the control and hypothyroid groups received sterile water as a placebo.

### Euthanasia and material collection

The animals were euthanized by decapitation on the 18th GD and blood was collected from the neck into tubes with heparin for dosage of free T3 and T4. The blood was centrifuged at 3000 rpm for 20 min and the plasma was obtained and stored at -20°C.

At necropsy, the entire genital system was collected. Subsequently, the uterus containing the placenta and fetuses, and the uterus with placenta were weighed, as were the fetuses, individually. Hysterectometric (maternal weight without the gravid uterus) maternal weight gain was also evaluated. The weight of amniotic fluid was estimated by subtracting the weight of the uterus and placenta with the fetuses from the weight of the fetuses and the weight of the uterus and placenta without the fetuses. The number of fetuses and the number of sites with resorption or fetal death were also counted. Fragments of the central region of the placenta measuring 2 mm in diameter were dissected and removed from two placental sites/animal and separately immersed in TRIzol^®^, followed by freezing in liquid nitrogen and storing at -80°C for quantitative real-time reverse transcriptase-polymerase chain reaction (qRT-PCR) analyses. The remaining discs (placenta+basal decidua+metrial gland) were fixed in 4% paraformaldehyde at 4°C for 24 hours and processed using the paraffin embedding technique for histomorphometry and immunohistochemistry analysis. The tissues were dehydrated in a serial solution of 70% to 100% alcohol, with subsequent xylene diaphanization and paraffin impregnation and embedding. Microtomy on histological slides was used to obtain 4-µm tissue sections for histomorphometry evaluation. Silane-coated polarized slides (StarFrost Polycat, Germany) were used for immunohistochemistry.

### Hormone analysis

Dosage of Free T3 and T4 was performed using enzyme-linked immunosorbent assay (ELISA) kits according to the manufacturer’s instructions (IMMULITE, Siemens Medical Solutions Diagnostics, Malvern, PA, USA).

### Evaluation of fetal development

The brain, heart, liver, lungs, and kidneys of each fetus were dissected and weighed, and the relative weight of the organs in relation to fetal weight was obtained. After weighing the fetal organs, the brain to liver ratio, an indicator of asymmetric fetal growth restriction, was calculated (47).

For analysis of fetal weight distribution and risk of fetal growth restriction, fetal weight histograms were constructed for each group with the individual fetal weight and non-linear regression was performed according to Dilworth et al. (48). The 5th percentile weight was calculated as: (-Z score x SD) + mean, assuming that Z score = 1.645, SD = standard deviation, mean = mean of control group.

### Histomorphometry analysis

Histomorphometry analysis of the placenta was performed on 4 µm histological sections stained with hematoxylin and eosin. All evaluations were performed blindly by two evaluators and without knowledge of the experimental groups. The quantitative evaluation was performed on 7-8 placental discs/group, and a histological section/placental disc was obtained from the center of the tissue with maternal central blood vessel to ensure the histological sections were uniform. Images of each placental disc were captured using a Leica S9i stereo microscope, and the thickness of each placental layer (junctional zone and labyrinth zone) was assessed in 10 random regions and averaged per placental site. The analyses were performed using Image Pro Plus*^®^* version 4.5 software and the values were transformed into millimeters using a micrometer scale.

In the junctional zone, the proportion of area occupied by glycogen cells, spongiotrophoblasts, and trophoblastic giant cells per field was evaluated by selecting 5 random fields with the 20x lens. In the labyrinth zone, the proportion of area occupied by maternal vascular sinus, fetal capillaries, and fetal mesenchyme/trophoblastic cells per field was evaluated by selecting 10 random fields of the labyrinth with the 40x lens. The images were captured on a Leica DM2500 photon microscope and quantification was performed using a graticule of 99 (junctional zone) and 100 (labyrinth zone) points with Image Pro Plus*^®^* software version 4.5 (35).

### Immunohistochemistry

Histological sections of the placental discs were submitted to immunohistochemistry analysis using the antibodies anti-8-OHdG (sc-393871), anti-HIF1α (sc-13515), anti-SOD1 (sc-101523), anti-catalase (sc-271803), anti-GPx 1/2 (sc-133160), anti-GRP78 (sc-13539), anti-CHOP (sc-71136), anti-ATF4 (sc-390063), anti-TNFα (sc-52746), anti-IL-10 (sc-365858), and anti-VEGF (sc-152), from Santa Cruz Biotechnology, CA, USA. All antibodies used in this study were validated by the manufacturer.

The streptavidin-biotin-peroxidase staining technique (Novolink Polymer Detection Systems, Leica Biosystems Inc., Buffalo Grove, IL, USA) was used and antigen retrieval was performed with heat in a water bath at 98°C using citric acid solution at pH 6.0. The slides were incubated in a humid chamber for 18 or 40 hours with the primary antibody (**Supplementary Figure 1**) and for 30 minutes in the blocking stages of endogenous peroxidase, serum blocking, and streptavidin peroxidase. The chromogen was diaminobenzidine (EnVision FLEX DAB+ Chromogen, Agilent Technologies, Inc., Santa Clara, CA. USA). The sections were counterstained with Harris hematoxylin. The negative control was obtained by replacing the primary antibody with phosphate buffered saline (PBS) (36).

A descriptive and quantitative evaluation of the immunohistochemistry expression of HIF1α, 8-OHdG, SOD1, Catalase, GPx1/2, GRP78, CHOP, ATF4, VEGF, TNFα, and IL-10 was performed in the junctional zone and labyrinth zone layers of the placenta. A quantitative evaluation was performed randomly on six placental discs/group. Images of 5 random fields in each region of the placental disc were obtained with a Leica DMI 300B photon microscope (Leica Microsystems, Germany) with the 40x lens. The immunolabeling area was determined using WCIF ImageJ^®^ software (Media Cybernetics Manufacturing, Rockville, MD, USA). The images were subjected to color deconvolution and thresholding. Data from each placental disc were archived, analyzed, and expressed as immunolabeling area in pixels (36).

### qRT-PCR

For the qRT-PCR technique, total RNA from the placenta was extracted using TRIzol^®^ according to the manufacturer’s instructions (Invitrogen, Life Technologies, Carlsbad, CA, USA). Subsequently, 1 µg of RNA was used for the reverse transcription reactions with the GoTaq^®^ qPCR and RT-qPCR Systems kit (A6010, PROMEGA). The transcripts of the target genes were quantified by qPCR using SYBR Green in the Applied Biosystems^®^ 7500 Real-Time PCR System. For the reactions, 1.5 μL of cDNA, 100 nM of each primer, and 12.5 μL of the GoTaq^®^ qPCR Master Mix 2X reagent was used in a final volume of 20 μL of reaction. As a negative control, the DNA amplification mix was used, in which the cDNA sample was replaced by water. The amplifications were performed under the following conditions: enzyme activation at 95°C for 2 min, 40 cycles of denaturation at 95°C for 15 s, and annealing/extension at 60°C for 60 s. To evaluate the linearity and efficiency of qPCR amplification, standard curves of all transcripts were generated using serial dilutions of the cDNA, followed by an evaluation of the melting curve of the amplification products. The primers for *Sod1*, *Catalase*, *Gpx1*, *Hif1α*, *Grp78*, *Chop*, *Il-10*, *Tnf*, *Il-6*, *Dio2*, *Igf1*, *Igf2*, *Glut1*, *Vegf*, and *Plgf* were delineated based on *Rattus norvegicus* mRNA sequence (**Table 1**). Gene expression was calculated by the 2^-ΔΔCT^ method, where the results obtained for each group were quantitatively compared after normalization based on the expression of *Polr2a Rattus norvegicus* (36, 49).

**Table 1:** List of genes and nucleotide sequences for qPCR primers.

Gene	Sequence (5'->3')	Accession number
*Sod1*	F: GAAAGGACGGTGTGGCCAATR: CTCGTGGACCACCATAGTACG	NM_017050.1
*Hifα*	F: AGCAATTCTCCAAGCCCTCCR: TTCATCAGTGGTGGCAGTTG	NM_024359.1
*Catalase*	F: CTGACTGACGCGATTGCCTAR: GTGGTCAGGACATCGGGTTT	NM_012520.2 R
*Gpx1*	F: GCGCTACAGCGGATTTTTGAR: GAAGGCATACACGGTGGACT	NM_030826.4
*Grp78*	F: TGAAGGGGAGCGTCTGATTGR: TCATTCCAAGTGCGTCCGAT	NM_013083.2
*Chop*	F: TGGCACAGCTTGCTGAAGAGR: TCAGGCGCTCGATTTCCT	NM_001109986.1
*Il10*	F: GGCCATTCCATCCGGGGTGAR: AAGGCAGCCCTCAGCTCTCG	NM_012854.2
*Tnfα*	F: AGCCCGTAGCCCACGTCGTAR: CGGTGTGGGTGAGGAGCACG	NM_012675.3
*Il6*	F: GACTTCCAGCCAGTTGCCTTR: AAGTCTCCTCTCCGGACTTGT	NM_053595.2
*Vegf*	F: GCCCAGACGGGGTGGAGAGTR: AGGGTTGGCCAGGCTGGGAA	NM_001110336.1
*Plgf*	F: CCGGCCCTGGCTGCATTGAAR: CAGGCAAAGCCCACAGGCGA	NM_053595.2
*Igf 1*	F: ACCCGGGACGTACCAAAATGR: CGAGCTGGTAAAGGTGAGCA	NM_178866.4
*Igf 2*	F: CATCATGTCCCACACTAAGGR: GTGCCAATTGGGTTGTTTAG	XM_008760074.2
*Dio2*	F: TGCAGTCTCATAGGTGCCTGGAAAR: ACCACTCCCAGCAACAACAACAAC	NM_031720.5
*Glut1 (Slc2a1)*	F: CAATCAAACATGGAACCACCGR: CGATTGATGAGCAGGAAGCG	NM_138827.1
*Polr2a*	F: GCTGGACCTACTGGCATGTTR: ACCATAGGCTGGAGTTGCAC	XM_001079162.5

### Statistical analysis

The differences of means among of the groups were determined by performing ANOVA followed by the Student-Newman-Keuls (SNK) test. The data were tested for normality (Shapiro-Wilk) and homoscedasticity (Brown-Forsythe) of the residuals, and for those that did not meet the assumptions, even after logarithmic transformation, the non-parametric Kruskal-Wallis test followed by Dunn’s test were used. Generalized linear mixed-model analysis followed by the Tukey test was used to evaluate the fetal and fetal organs’ weight and liver/brain ratio (50). Parametric data were represented by mean ± standard error of the mean (SEM), while non-parametric data were represented by median and interquartile range. The analyses were performed using GraphPad Prism 9.0.0 and R version 4.2.0 software, and differences where P<0.05 were considered significant.

## Results

### Daily kisspeptin treatment improves fetal restricted growth and plasma free T3 and T4 levels in hypothyroid rats

**Figure 1:**
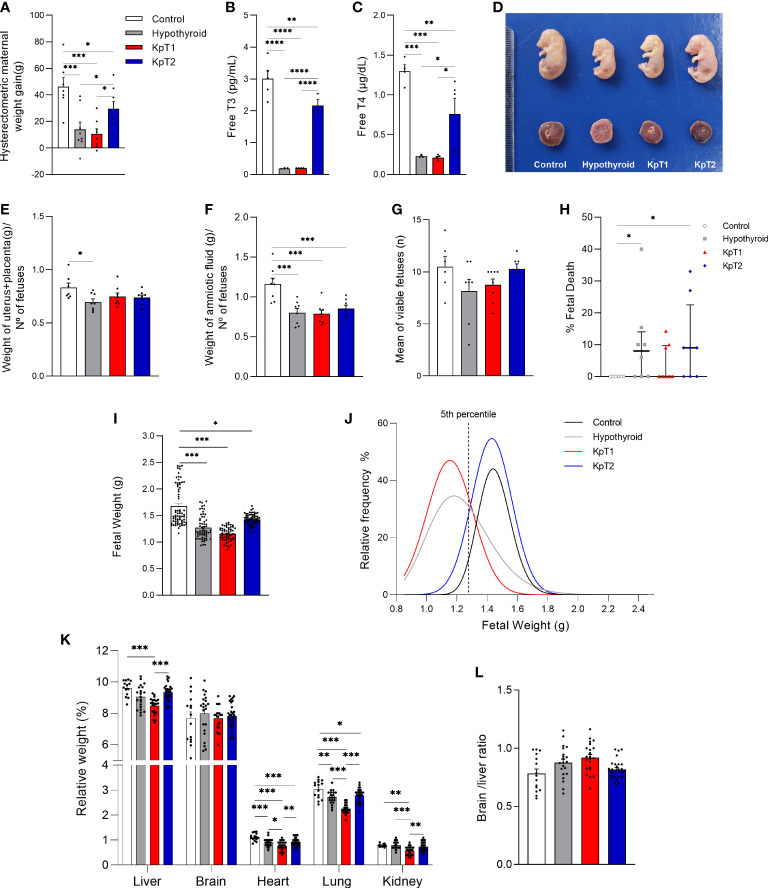
Maternal and fetal parameters of control, hypothyroid, and kisspeptin-10-treated rats on the 18th GD. **(A)** Hysterectometric maternal weight gain (mean ± SEM; n = 8). **(B)** Free T3 (mean ± SEM; n = 4-5). **(C)** Free T4 (mean ± SEM; n = 4-5). **(D)** Representative image of fetuses and placentas from each experimental group … **(E–F)** Uteroplacental **(E)** and amniotic fluid **(F)** weight (mean ± SEM; n = 8). **(G)** Number of viable fetuses/litter (mean ± SEM; n = 8). **(H)** Percentage of fetal death (median, interquatile range; n = 8). **(I)** Fetal weight (mean ± SEM; n = 63-76). **(J)** Relative frequency distribution curve of fetal weight. **(K)** Weight of fetal organs (liver, brain, heart, lung, kidney) (mean ± SEM; n = 15-30). **(L)** Brain/liver ratio (mean ± SEM; n = 15-30). Significant differences were determined by ANOVA *post hoc* SNK except for fetal weight and fetal organs’ weight, which were determined by Generalized linear mixed-model analysis followed by the Tukey test, and fetal death which was determined by Dunn’s *post hoc* Kruskal-Wallis test, *P<0.05, **P<0.01, ***P<0.001, ****P<0.0001. KpT1, yes/no day treatment with Kp10; KpT2, daily treatment with Kp10; GD, gestational day.

Because fetal growth restriction in hypothyroid rats is associated with reduced Kiss1 and Kiss1R expression at the maternal-fetal interface (31), the effect of two treatment protocols with Kp-10 was evaluated every other day (KpT1) and daily (KpT2), starting on the 8th GD, on the maternal and fetal parameters of hypothyroid rats. The evaluation of maternal and placental parameters revealed that daily treatment with Kp-10 increased hysterectometric maternal weight gain during gestation when compared to the hypothyroid and KpT1 groups (**Figure 1A**; P<0.05), which had lower gestational weight gain compared to the control (P<0.001). No significant difference was observed between the hypothyroid and KpT1 groups for hysterectometric maternal weight gain (P>0.05). Interestingly, there was an increase in plasma free T3 and T4 levels in the group treated daily with Kp10 compared to the hypothyroid and KpT1 groups (**Figures 1B, C**; P<0.0001; P<0.05) which had lower free T3 and T4 levels compared to the control (P<0.001). Furthermore, the KpT1 and KpT2 treatments did not increase the uteroplacental weight or the amniotic fluid of the hypothyroid animals (P>0.05), which showed a reduction compared to the control group (P<0.05) (**Figures 1E, F**).

In the evaluation of fetal parameters, although a significant difference was not observed in the number of viable fetuses between the experimental groups (**Figure 1G**), the KpT1 and KpT2 treatments did not present any differences in the percentage of fetal death when compared to the hypothyroid group (P>0.05), which showed a higher percentage of fetal death when compared to the control (**Figure 1H**; P<0.05). The KpT1 and KpT2 treatments were also unable of restore the reduction of fetal weight cause by hypothyroidism when compared to the control (**Figures 1D, I**; P<0.001; P<0.05). However, in assessing the relative frequency of fetal weight distribution, both the KpT2 (87.30%) and control (95.05%) groups had a greater distribution than 5th percentile (1.278g) (dashed line), while most fetuses in the hypothyroid (61.49%) and KpT1 (78.58%) groups was below the 5th, with a shift to the left of the distribution curve (**Figure 1J**).

The KpT2 treatment did not restore the lower heart and lung weights exhibited by the fetuses of hypothyroid animals (P>0.05), which had reduced weights when compared to the control (**Figure 1K**; P<0.001; P<0.01). However, the fetuses in the KpT1 group had lower liver weight when compared to the control and KpT2 groups (P<0.001), as well as lower heart and lung weight in relation to the control and hypothyroid groups. Regarding kidney weight, the fetuses of the KpT1 group also showed a reduction compared to the control (P<0.01), hypothyroid (P<0.001), and KpT2 (P<0.01) groups. No significant difference was observed for fetal brain weight and the brain/liver ratio between the experimental groups (**Figures 1K, L**; P>0.05). Together, these data demonstrate that daily treatment with Kp10 was not able to increase fetal weight, but improved the “growth restricted” condition demonstrated by the fetal weight distribution curve. This was unlike the KpT1 treatment, which did not increase fetal weight, kept the greater part of the fetal weight below the 5th percentile of the control group, and had negative effects on fetal organogenesis.

### Kisspeptin treatment improves placental development in hypothyroid rats

**Figure 2:**
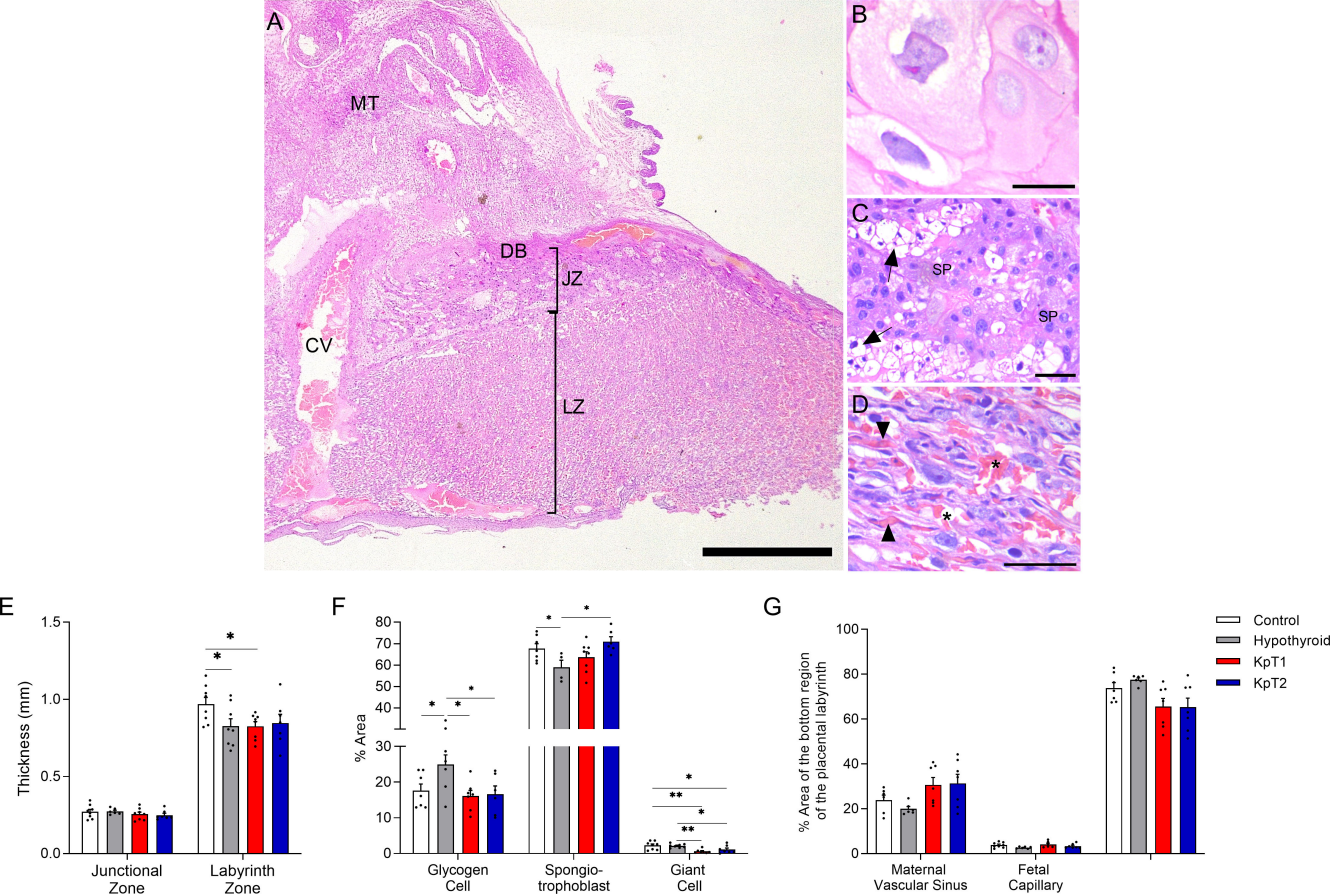
Histomorphometry evaluation of the placenta of control, hypothyroid, and kisspeptin-10 treated-rats on the 18th GD. **(A–D)** Illustrative photomicrographs of the maternal-fetal interface **(A)** and placental layers **(B)**, giant cells; **(C)**, spongiotrophoblast; **(D)**, labyrinth zone) (Hematoxylin and eosin staining; Bar = 500μm **(A)**; 50μm **(B–D). (E)** Thickness of the junctional zone (giant cells+spongiotrophoblast) and labyrinth zone (mean ± SEM; n = 8). **(F)** Percentage of area occupied by glycogen cells, spongiotrophoblasts, and giant cells in the junctional zone (mean ± SEM; n = 8). **(G)** Percentage of area occupied by maternal vascular sinus, fetal capillaries, and fetal mesenchyme/trophoblast in the labyrinth zone (mean ± SEM; n = 8). Significant differences were determined by ANOVA *post hoc* SNK, *P<0.05, **P<0.01. MT, mesometrial triangle; BD, basal decidua; JZ, junctional zone; LZ, labyrinth zone; CV, central vessel; SP, spongiotrophoblast; Arrow, glycogen cells; Arrowhead, fetal capillaries; Asterisks, maternal vascular sinus; KpT1, yes/no day treatment with Kp-10; KpT2, daily treatment with Kp-10; GD, gestational day.

After observing that daily treatment with Kp-10 improved fetal development in hypothyroid rats, it was necessary to assess whether this effect could result from improved placental morphology (**Figures 2A–D**). No difference was observed in the thickness of the junctional zone between the experimental groups, while the hypothyroid and KpT1 groups showed reduced thickness of the labyrinth zone when compared to the control group (**Figure 2E**; P<0.05). In the cellularity evaluation of the junctional zone, the placenta of hypothyroid animals had a higher percentage of area occupied by glycogen cells and fewer spongiotrophoblasts when compared to the control group (P<0.05). The treatments with KpT1 and KpT2 were able to restore the population of glycogen cells and/or spongiotrophoblasts in the placenta of hypothyroid animals, resembling that of the control animals (**Figure 2F**; P>0.05). A significant reduction was also observed in the giant cells from junctional zone in the KpT1 and KpT2 groups compared to the control (P<0.01;P<0.05) and to the hypothyroid group (**Figure 2F**; P<0.01;P<0.05). In the evaluation of the labyrinth zone, no significant difference was observed in the regions evaluated (fetal capillary, maternal vascular sinus and fetal mesenchyme/trophoblastic cells) between the groups (**Figure 2G**; P>0.05).

### Kisspeptin treatment increases placental gene expression of growth (*Plgf*, *Igf1*) and transport (*Glut1*) factors in rats with hypothyroidism

The macroscopic evaluations of fetal-placental development and histological evaluations of the placenta showed that daily treatment with Kp10 had a better effect on fetal and placental development of hypothyroid rats, particularly by improving fetal weight distribution and junctional zone cellularity, while the group KpT1 presented negative effects on fetal organogenesis. For this reason, the placental expression of growth (VEGF, PLGF, IGF1, IGF2) and hormonal (Dio2) factors, and transporters (GLUT1) was evaluated in the KpT2 group in relation to the control and hypothyroid groups, as the altered expression of these factors is associated with altered fetal-placental growth (51–54).

**Figure 3:**
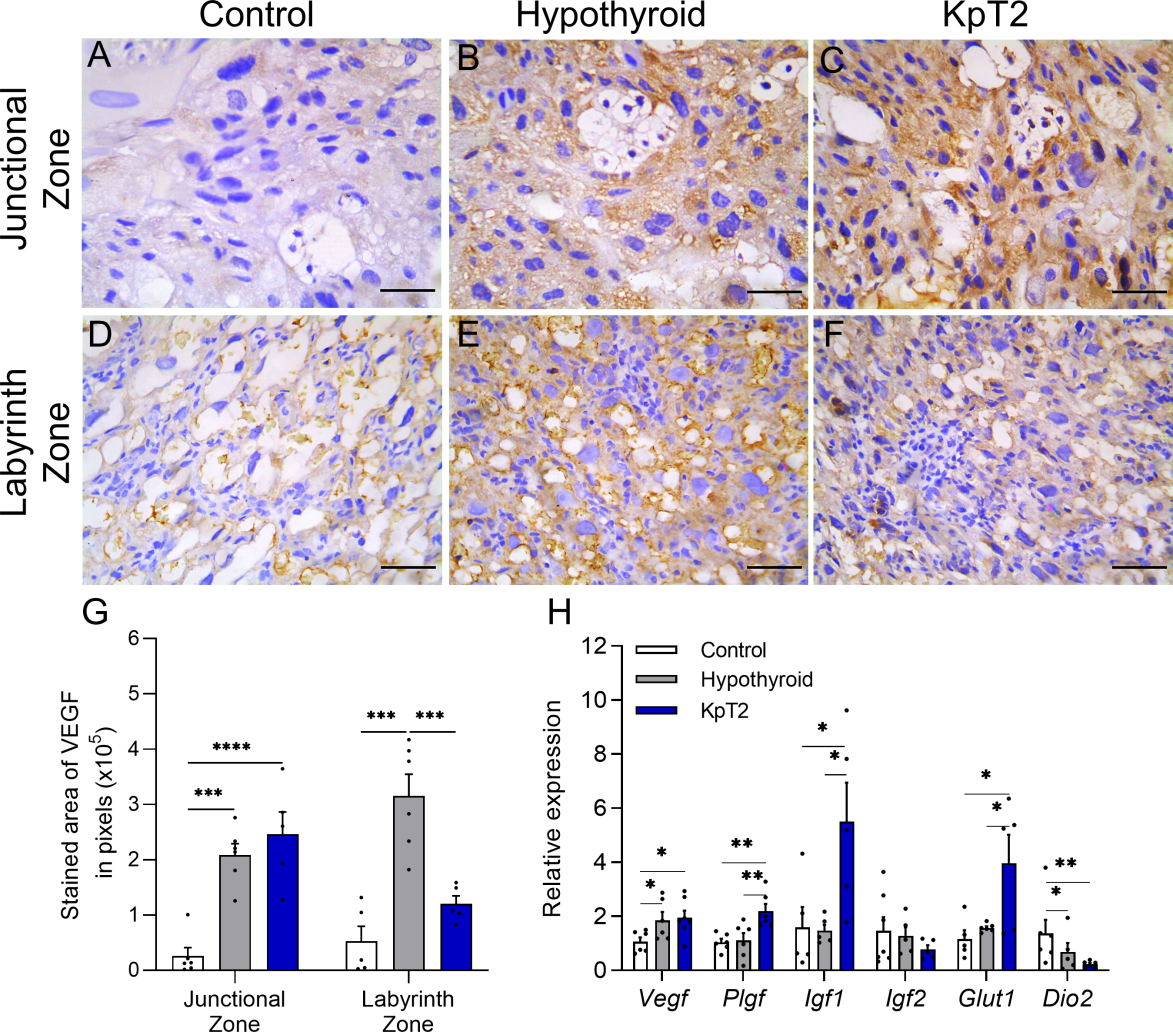
Expression of VEGF, PIGF, IGF1, IGF2, GLUT1 and DIO2 in the placenta of control, hypothyroid, and kisspeptin-10-treated rats on the 18th GD. **(A–F)** Photomicrographs of immunohistochemical expression of VEGF in the junctional zone **(A–C)** and labyrinth zone **(D–F)** (Streptavidin-biotin-peroxidase; Harris hematoxylin; Bar = 50 µm). **(G)** Immunolabeling area, in pixels, of VEGF expression in the junctional zone and labyrinth zone on the 18 GD (mean ± SEM; n = 8). **(H)** Relative gene expression of *Vegf, Pigf, Igf1, Igf2*, *Glut1, and Dio2* in the placenta (mean ± SEM; n = 8). Significant differences were determined ANOVA post hoc SNK, *P<0.05, **P<0.01, ***P<0.001, ****P<0.0001. KpT2, daily treatment with Kp10; GD, gestational day.

Immunolabeling for VEGF was cytoplasmic and heterogeneous in trophoblasts in the junctional and labyrinth zones, regardless of the experimental group. However, the analysis of the labeled area revealed that the hypothyroid and KpT2 groups had higher expression of VEGF in the junctional zone when compared to the control group (**Figures 3A–C, G**). This was also observed in the placental gene transcript expression for *Vegf*, which had higher expression in the hypothyroid and KpT2 groups compared to the control group (**Figure 3H**; P<0.05). In the labyrinth zone, on the other hand, KpT2 treatment reduced VEGF immunolabeling compared to the hypothyroid group (P<0.001), equaling the control group (**Figures 3D–G**; P>0.05). Regarding the other placental growth and transport factors, daily treatment with Kp10 significantly increased mRNA expression for *Plgf, Igf1*, and *Glut1* in the placenta when compared to the control and hypothyroid groups (**Figure 3H**; P<0.05; P<0.01). For *Igf2*, no difference was observed between the groups (P>0.05), while *Dio2* had lower expression in the hypothyroid and KpT2 groups compared to the control group (**Figure 3H**; P<0.01; P<0.01).

### Kisspeptin treatment alters placental IL10 and TNFα expression in rats with hypothyroidism

**Figure 4:**
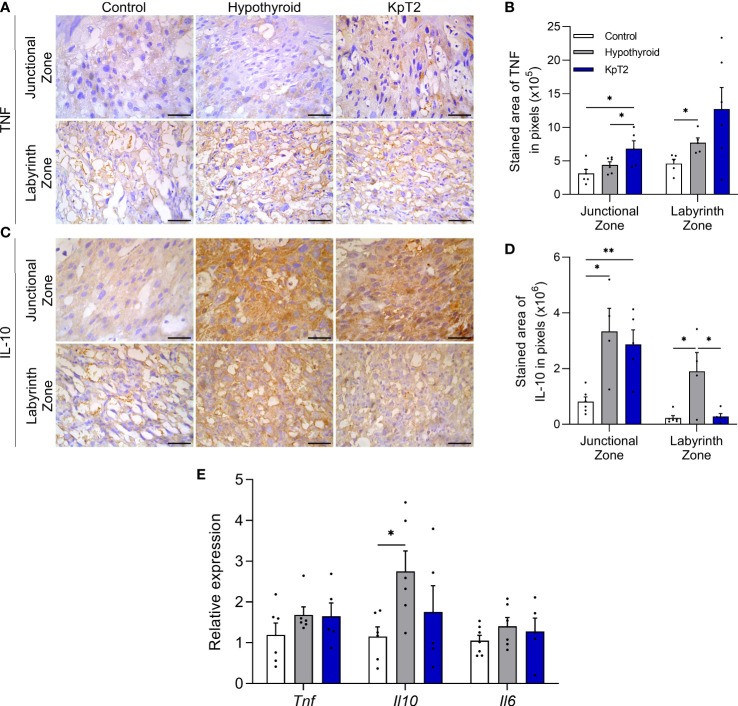
Expression of TNFα, IL-10, and IL-6 in the placenta of control, hypothyroid, and kisspeptin-10-treated rats on the 18th GD. **(A)** Photomicrographs of immunohistochemical expression of TNFα in the junctional zone and labyrinth zone (Streptavidin-biotin-peroxidase; Harris hematoxylin; Bar = 50 µm). **(B)** Immunolabeling area, in pixels, of TNFα expression in the junctional zone and labyrinth zone (mean ± SEM; n = 8). **(C)** Photomicrographs of immunohistochemical expression of IL-10 in the junctional zone and labyrinth zone (Streptavidin-biotin-peroxidase; Harris hematoxylin; Bar = 50 µm). **(D)** Immunolabeling area, in pixels, of IL-10 expression in the junctional zone and labyrinth zone (mean ± SEM; n = 8). **(E)** Relative gene expression of *Tnf, Il10*, and *Il6* in the placenta (mean ± SEM; n = 8). Significant differences were determined by ANOVA *post hoc* SNK, *P<0.05, **P<0.01. KpT2, daily treatment with Kp10; GD, gestational day.

Since fetal-placental development is influenced by immune mediators produced by the placenta, and considering that previous studies have shown that maternal hypothyroidism affects placental TNFα, MIF, and NOS2 expression in rats (36), the effect of daily treatment with Kp-10 on placental TNFα, IL-10, and IL-6 expression was evaluated in hypothyroid rats. TNFα and IL-10 immunolabeling was cytoplasmic in trophoblastic cells of the junctional and labyrinth zones, and was heterogeneous for TNFα and homogeneous for IL-10 (**Figures 4A, C**). Immunolabeling for TNFα was more intense in the junctional zone of the Kp10 group than in the control and hypothyroid groups (**Figure 4A**), as confirmed by the immunolabeling area analysis (**Figure 4B**; P<0.05). In the labyrinth zone, hypothyroid rats showed higher TNFα expression compared to the control (P<0.05), while the Kp10 treatment was similar to the hypothyroid group (P>0.05). No significant differences in mRNA expression for *Tnf* were observed between the groups (**Figure 4E**; P>0.05).

For IL-10, both the hypothyroid and KpT2 groups showed higher immunolabeling in the junctional zone compared to the control (**Figure 4C**), as confirmed by the immunolabeling area analysis (**Figure 4D**; P<0.05; P<0.01). In the labyrinth zone, however, while hypothyroidism also increased the IL-10 labeling area when compared to the control (P<0.05), Kp10 treatment reduced IL-10 immunolabeling, matching it to that of the control group (**Figure 4D**; P>0.05). As observed in the immunohistochemistry, hypothyroidism increased mRNA expression for *Il10* compared to the control (P<0.05), while daily treatment with Kp10 showed no significant differences compared to the control and hypothyroid groups (**Figure 4E**; P>0.05). There was no difference in the mRNA expression for *Il6* between the groups (**Figure 4E**).

### Kisspeptin treatment blocks oxidative damage in the junctional zone of hypothyroid rats

Since oxidative stress is associated with placental dysfunction (55–57), and maternal hypothyroidism has been recently reported to cause oxidative damage in rat placenta (40), it was important to evaluate whether daily treatment with Kp10 can reverse or reduce this oxidative stress caused by hypothyroidism. To this end, the gene and/or protein expression profile of HIF1α and 8-Hydroxy-2′-deoxyguanosine (8-OHdG), biomarkers of hypoxia and oxidative DNA damage, respectively (58–60), were analyzed.

**Figure 5:**
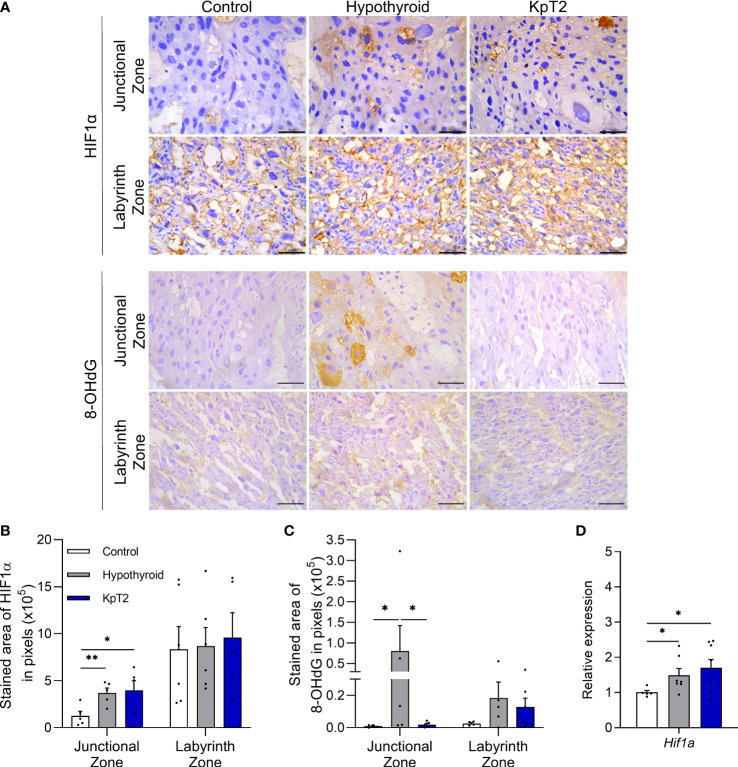
Expression of HIF1α and 8-OHdG in the placenta of control, hypothyroid, and kisspeptin-10-treated rats on the 18th GD. **(A)** Photomicrographs of immunohistochemical expression of HIF1α in the junctional zone and labyrinth zone (Streptavidin-biotin-peroxidase; Harris hematoxylin; Bar = 50 µm). **(B)** Immunolabeling area, in pixels, of HIF1α expression in the junctional zone and labyrinth zone (mean ± SEM; n = 8). **(C)** Immunolabeling area, in pixels, of HIF1α expression in the junctional zone and labyrinth zone (mean ± SEM; n = 8). **(D)** Relative gene expression of *Hif1α* in the placenta (mean ± SEM; n = 8). Significant differences were determined by ANOVA *post hoc* SNK, *P<0.05, **P<0.01. KpT2, daily treatment with Kp10; GD, gestational day.

HIF1α and 8-OHdG immunolabeling were cytoplasmic and heterogeneous in trophoblastic cells of the junctional and labyrinth zones, regardless of the experimental group (**Figures 5A, B**). In the HIF1α immunolabeling analysis, both the hypothyroid and Kp10-treated groups showed a larger immunolabeling area in the junctional zone when compared to the control (**Figures 5C**, P<0.05). In the labyrinth zone, in contrast, no significant differences in immunolabeling were observed between the groups (P>0.05). However, in gene evaluation, similar to the result of junctional zone immunolabeling, the hypothyroid and Kp10-treated groups showed higher placental mRNA expression of *Hif1α* compared to the control (**Figure 5E**; P<0.05).

Regarding 8-OHdG immunolabeling, Kp10 treatment reduced the greater immunolabeling in the junctional zone caused by hypothyroidism (P<0.05), equaling the control (**Figure 5D**; P >0.05). No difference was observed in 8-OHdG expression in the labyrinth zone between the groups (P>0.05).

### Kisspeptin treatment increases placental expression of antioxidant enzymes in hypothyroid rats

**Figure 6:**
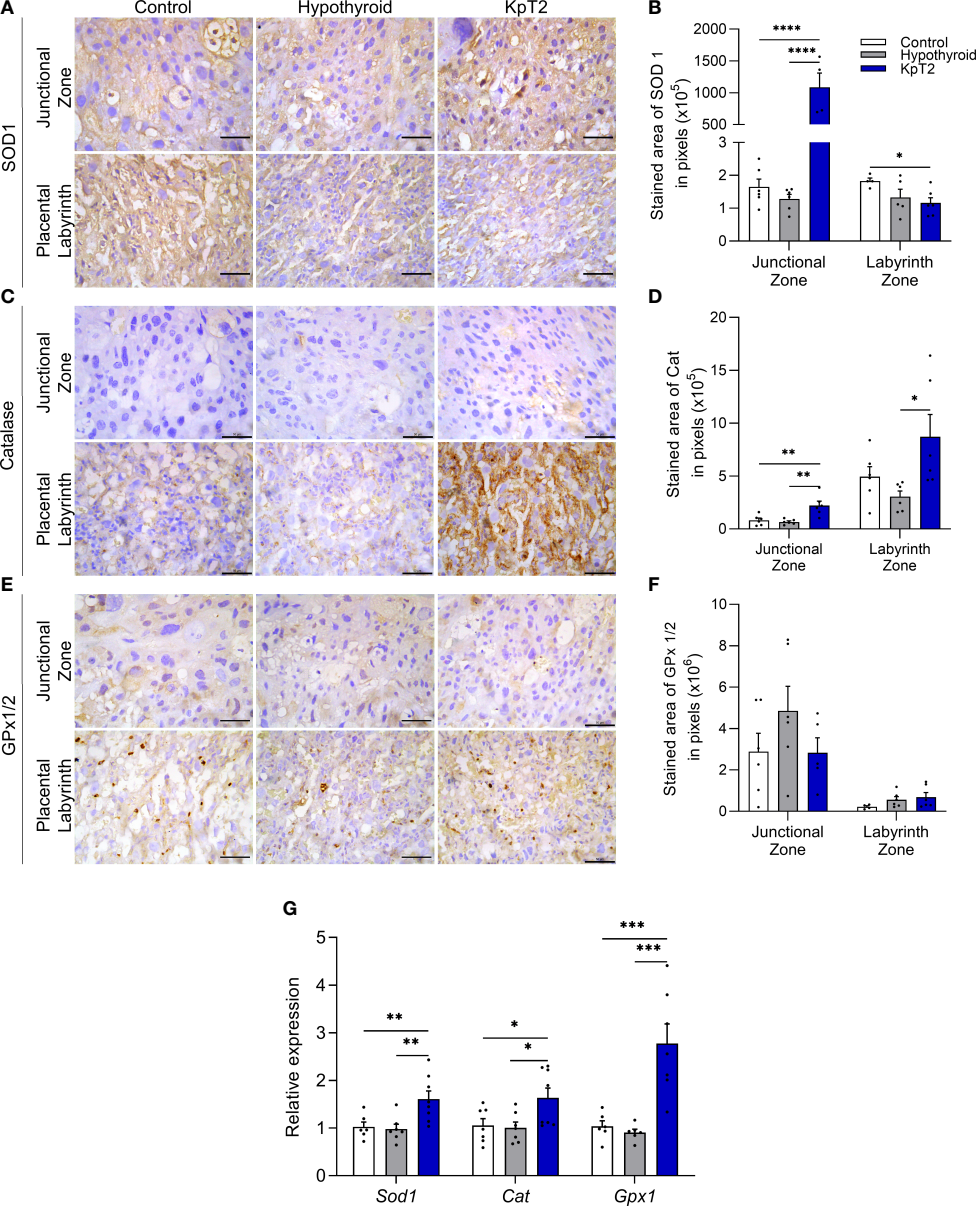
Expression of SOD1, catalase, and GPx1/2 in the placenta of control, hypothyroid, and kisspeptin-10-treated rats on the 18th GD. **(A)** Photomicrographs of immunohistochemical expression of SOD1 in the junctional zone and labyrinth zone (Streptavidin-biotin-peroxidase; Harris hematoxylin; Bar = 50 µm). **(B)** Immunolabeling area, in pixels, of SOD1 expression in the junctional zone and labyrinth zone (mean ± SEM; n = 8). **(C)** Photomicrographs of immunohistochemical expression of catalase in the junctional zone and labyrinth zone (Streptavidin-biotin-peroxidase; Harris hematoxylin; Bar = 50 µm). **(D)** Immunolabeling area, in pixels, of catalase expression in the junctional zone and labyrinth zone (mean ± SEM; n = 8). **(E)** Photomicrographs of the immunohistochemical expression of GPx1/2 in the junctional zone and labyrinth zone (Streptavidin-biotin-peroxidase; Harris hematoxylin; Bar = 50 µm). **(F)** Immunolabeling area, in pixels, of GPx1/2 expression in the junctional zone and labyrinth zone (mean ± SEM; n = 8). **(G)** Relative gene expression of *Sod1*, *catalase*, and *Gpx1* in the placenta (mean ± SEM; n = 8). Significant differences were determined by ANOVA *post hoc* SNK, *P<0.05, **P<0.01, ***P<0.001, ****P<0.0001. KpT2, daily treatment with Kp10; GD, gestational day.

Since studies have already demonstrated that exogenous administration of kisspeptin exhibits antioxidant action in the ovary (43), liver (44), and testicle (45), the placental profile of SOD1, catalase, and GPx1/2 was evaluated for the animals in the present study. The immunostaining of SOD1, catalase, and GPx1/2 was cytoplasmic and heterogeneous in trophoblast cells of the junctional and labyrinth zones, and the expression of catalase was weaker in the junctional zone when compared to that of SOD1 and GPx1/2 (**Figures 6A, C** and **E**). Treatment with Kp10 significantly increased the expression of SOD1 and catalase in the junctional zone when compared to the control and hypothyroid groups (**Figures 6B, D**; P<0.01, P<0.0001). In the labyrinth zone, while the KpT2 group showed reduced SOD1 immunolabeling compared to the control (P<0.05), Kp10 treatment increased catalase expression when compared to the hypothyroid group (**Figures 6B, D**; P<0.05). No significant difference was observed in the GPx1/2 immunolabeling between groups in both the junctional zone and labyrinth zone (**Figure 6F**; P>0.05). However, in the gene transcript expression analysis, *Gpx1*, *Sod1*, and *Cat* showed higher mRNA expression in the Kp10-treated group when compared to the control and hypothyroid groups (**Figure 6G**).

### Kisspeptin treatment affects the expression of reticular stress mediators in the placenta of hypothyroid rats

**Figure 7:**
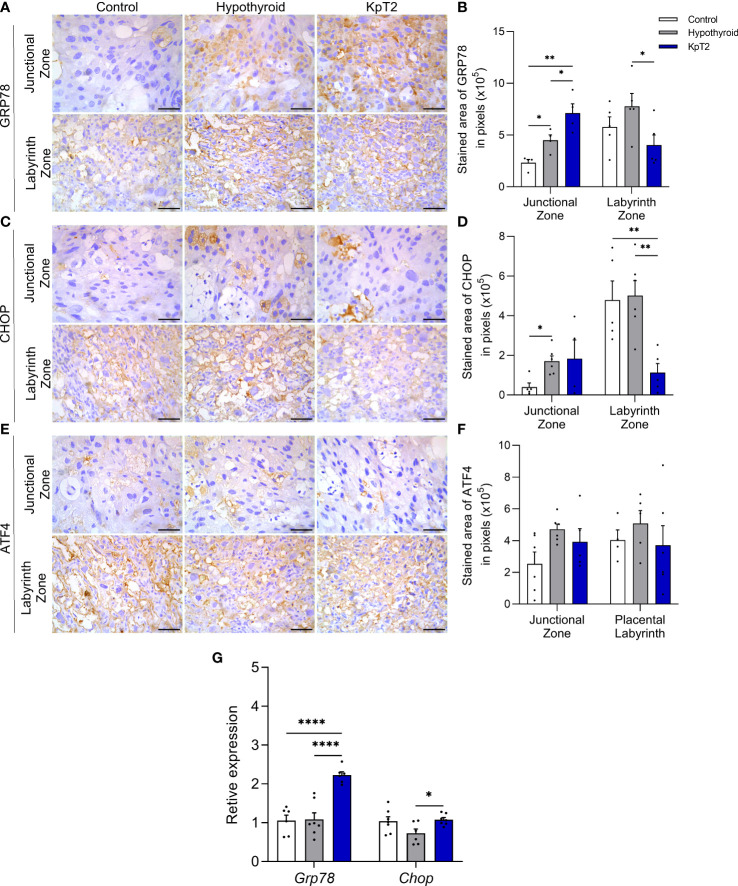
Expression of GRP78, CHOP, and ATF4 in the placenta of control, hypothyroid, and kisspeptin-10-treated rats on the 18th GD. **(A)** Photomicrographs of immunohistochemical expression of GRP78 in the junctional zone and labyrinth zone (Streptavidin-biotin-peroxidase; Harris hematoxylin; Bar = 50 µm). **(B)** Immunolabeling area, in pixels, of GRP78 expression in the junctional zone and labyrinth zone (mean ± SEM; n = 8). **(C)** Photomicrographs of immunohistochemical expression of CHOP in the junctional zone and labyrinth zone (Streptavidin-biotin-peroxidase; Harris hematoxylin; Bar = 50 µm). **(D)** Immunolabeling area, in pixels, of CHOP expression in the junctional zone and labyrinth zone (mean ± SEM; n = 8). **(E)** Photomicrographs of immunohistochemical expression of ATF4 in the junctional zone and labyrinth zone (Streptavidin-biotin-peroxidase; Harris hematoxylin; Bar = 50 µm). **(F)** Immunolabeling area, in pixels, of ATF4 expression in the junctional zone and labyrinth zone (mean ± SEM; n = 8). **(G)** Relative gene expression of *Grp78* and *Chop* in the placenta (mean ± SEM; n = 8). Significant differences were determined by ANOVA *post hoc* SNK, *P<0.05, **P<0.01, ****P<0.0001. KpT2, daily treatment with Kp10; GD, gestational day.

Since maternal hypothyroidism causes reticular stress in rat placenta (40), and *in vitro* and *in vivo* studies have demonstrated that Kp-10 administration blocks the occurrence of reticular stress in hypothalamic neuronal cells exposed to androgen (46), it was evaluated whether Kp-10 treatment would affect the expression of GRP78, CHOP and ATF4 in the placenta of rats with hypothyroidism. Immunolabeling of these three mediators was cytoplasmic and heterogeneous in the junctional and labyrinth zones, and expression in the junctional zone was usually in isolated spongiotrophoblast cells or in glycogen cells (**Figures 7A, C** and **E**). According to the immunolabeling area analysis, the hypothyroid group showed higher immunolabeling of GRP78 and CHOP in the junctional zone when compared to the control (**Figures 7B, D**; P<0.05), while the KpT2 group showed no significant difference compared to the hypothyroid group for CHOP. However, with respect to GRP78, Kp-10 treatment increased immunolabeling in the junctional zone when compared to the control and hypothyroid groups (**Figure 7B**; P<0.05; P<0.01). In contrast, in the labyrinth zone, KpT2 treatment reduced the immunolabeled area of both GRP78 and CHOP relative to the hypothyroid group (**Figures 7B, D**; P<0.05; P<0.01). For ATF4, no differences in the immunolabeling area were observed between the groups (**Figures 7F**; P>0.05).

In the gene transcript analysis, treatment with Kp-10 increased the placental expression of *Grp78* compared to the control and hypothyroid groups (P<0.0001), as observed in junctional zone immunolabeling, while an increase in *Chop* gene expression was also observed relative to the hypothyroid group (**Figure 7G**; P<0.05). Taken together, these results demonstrate that daily treatment with Kp10 differentially affects the expression of reticular stress mediators in the placenta of hypothyroid rats since it maintains high expression in the junctional zone and reduces immunolabeling in the labyrinth zone.

## Discussion

Although the kisspeptin/Kiss1R system is mainly known for its action on the hypothalamic-pituitary-gonadal axis, stimulating the secretion of GnRH/LH, studies have shown that kisspeptin has peripheral action in the genital tract (5, 6). and gestational dysfunctions such as preeclampsia, spontaneous abortion, gestational diabetes, obesity, and even maternal hypothyroidism, are associated with alterations in the serum and/or placental profile of kisspeptin (12, 17, 20, 26–28, 30, 31). The results of the present study demonstrated that daily kisspeptin treatment was able to improve fetal development and placental morphology in hypothyroid rats. This improvement in fetal-placental development was associated with both increased expression of growth and transport factors in the placenta *(Plgf*, *Igf1*, and *Glut1*), and with blockage of placental oxidative damage and increased antioxidant enzymes (SOD1, catalase, GPx1).

Daily treatment with Kp-10 from the 8th GD not only increased maternal weight gain and plasma free T3 and T4 levels of hypothyroid rats, but also improved fetal weight distribution, which was not observed in the treatment performed every other day. These results reaffirm the need for precise control of kisspeptin plasma levels during pregnancy, since only daily treatment with Kp-10 had a positive effect on maternal weight gain, fetal weight distribution and on free T3 and T4 levels. This is the first *in vivo* study to demonstrate that kisspeptin can modulate the Corroborating our study, Radwańska and Kosior-Korzecka (61) demonstrated that *in vitro* treatment of ovine pituitary cells with 10^−11^ to 10^−8^ M of Kp10 increased TSH secretion. However, despite the increase in plasma levels of free T3 and T4 caused by the daily treatment with Kp-10, it is suggested that the local availability of T3 in the placenta has not changed, since the treatment with Kp-10 did not change the lower placental expression of *Dio2* caused by hypothyroidism, the enzyme responsible for the intracellular availability of T3 (62). This reduction of *Dio2* in the placenta of the hypothyroid rats may be associated with fetoplacental dysfunction presented by these animals, since *Dio2* is involved in proliferation, differentiation and trophoblastic migration and low placental expression was associated early recurrent miscarriage (54).

The increase in the maternal weight gain and improviment fetal weight distribution of hypothyroid rats may be associated not only with increased levels of free T3 and T4 but also with the action of kisspeptin on maternal pancreatic function, as exogenous kisspeptin increases *in vivo* and *in vitro* insulin secretion by pancreatic β-cells in rats, mice, humans, and non-human primates (63–67), and pharmacological blockade or *in vivo* genetic ablation of the Kiss1R receptor in β-cells of pregnant mice results in glucose intolerance and impaired insulin secretion (68).

For fetal weight distribution, most fetuses (61.49%) of the hypothyroid group were below the 5th percentile of the control group, indicating the growth restriction (48, 50, 69, 70). Once the reduced body weight was accompanied by reduced fetal organ weight (heart and lung), prioritizing the brain development, we suggest the growth restriction caused by hypothyroidism was asymmetric, even though was no difference in the liver/brain ratio (48, 50, 71). Daily treatment with Kp-10 improved fetal weight distribution once most of the fetal weight (87.30%) were above the 5th percentile of the control group. This outcome in terms of fetal development is very important, as fetal growth restriction is associated with an increased risk of cardiovascular and metabolic diseases in postnatal life (51, 72).

However, optimal fetal development depends not only on maternal and fetal metabolism but also on adequate placental function (32). In the present study, daily treatment with Kp10 also improved the placental morphology of hypothyroid rats by restoring the proportion of trophoblastic cells forming the junctional zone. Previous studies have also demonstrated altered trophoblast population in the junctional zone of hypothyroid rats (35). The cells in the junctional zone are mainly responsible for the synthesis and secretion of peptides and hormones by the placenta, which are fundamental for controlling maternal and fetal metabolism (73).

In addition to improving placental morphology, daily treatment with Kp-10 increased mRNA expression for *Plgf, Igf1*, and *Glut1* in the placenta. These factors are decisive for proper fetal growth and placental development (32, 51, 74, 75). PLGF is responsible for placental vascular bed maturation (76), and low plasma PLGF levels are associated with fetal growth restriction, HELLP syndrome, preeclampsia, and gestational hypertension (74). Therefore, plasma PLGF is considered an important predictor of fetal development (74). Reduced placental expression of IGF1 also compromises fetal and placental development (75), while GLUT1 is an important placental glucose transporter. The expression of GLUT1 is reduced when the placental expression of IGF1 fails (77, 78).Therefore, in the present study, restored fetal weight distribution caused by Kp-10 treatment may have also resulted from increased placental IGF1/GLUT1 signaling, as kisspeptin stimulates the release of insulin (63–68).

However, in relation to VEGF, treatment with Kp-10 did not reverse the increase in placental gene and protein expression caused by hypothyroidism, particularly in the junctional zone. In addition to the critical role of VEGF in the process of placental angiogenesis, especially in early to mid-gestation (79), it is an indicator of hypoxia since low oxygen levels stimulate its expression (80). In this sense, higher gene expression of VEGF in the placenta of hypothyroid and Kp-10-treated animals, and the higher immunolabeling in the junctional zone, suggest a low oxygen tension environment in the junctional zone of these animals. This hypothesis is supported by the higher gene and protein expression of HIF1α, especially in the junctional zone, observed in the hypothyroid and Kp-10-treated animals. HIF1α is expressed under hypoxic conditions and performs its functions in vascular permeability and cell survival by signaling *via* VEGF (81). Furthermore, studies have already demonstrated increased expression of HIF1α and VEGF in preeclamptic placentas, which are known to present a hypoxic environment (81–83). However, when evaluating the labyrinth zone, Kp10 treatment reduced the increase in VEGF immunolabeling caused by hypothyroidism, matching its expression to that of the control animals. These data reinforce the importance of immunohistochemistry analysis to discriminate and adequately evaluate the protein expression profile of the mediators evaluated in each region of the placenta.

Regarding TNFα, Kp10 treatment increased immunolabeling in the junctional zone, while in the labyrinth zone, hypothyroid animals showed higher expression compared to the control animals. *In vitro* studies have shown that TNFα secreted by decidual macrophages is recognized by TNF-R1 receptors in the trophoblasts and activates the extrinsic apoptosis pathway (84), while, according to a recent study, kisspeptin increases cell apoptosis (85). Therefore, Kp-10 treatment may have increased activation of placental apoptosis *via* TNFα signaling, although studies are needed to confirm this hypothesis. However, increased TNFα also occurs with increasing insulin resistance (86). Thus, another possibility to explain its higher expression in the junctional zone of Kp-10-treated animals may be the increase in insulin that occurs after kisspeptin administration (63–68).

As observed for TNFα, IL-10 showed higher expression in the junctional zone of hypothyroid and Kp-10-treated animals compared to the control, as well as higher gene expression in the placenta of hypothyroid animals. This increase in IL-10 may be a reflection of increased TNFα expression, as IL-10 attenuates the effects of pro-inflammatory cytokines at the maternal-fetal interface (38, 87) and TNFα stimulates the *in vitro* secretion of IL-10 in first-trimester trophoblastic villi (88). However, Kp-10 treatment reduced the higher IL-10 immunolabeling in the labyrinth zone caused by hypothyroidism, matching it to that of the control. Further studies are needed to elucidate the reduction of IL10 and VEGF in the labyrinth zone of the hypothyroid rats caused by Kp10 treatment.

As previously described, the increased mRNA and immunolabeling of HIF1α in the junctional zone of hypothyroid and Kp-10-treated animals signals low oxygen tension and suggests the occurrence of oxidative stress in the placenta of hypothyroid rats, which was recently confirmed in a previous study (40). Activation of HIF1α in the placenta of rats also favors the reduction of labyrinth zone thickness (89), thus corroborating the histomorphometry results of the present study. Moreover, Kp-10 treatment blocked the increase in placental immunolabeling of 8-OHdG caused by hypothyroidism. 8-OHdG is a biomarker of oxidative stress that signals DNA damage resulting from oxidative stress and lipid peroxidation (90), and studies have shown increased placental expression in preeclampsia, gestational diabetes mellitus, and maternal smoking (60, 90, 91). Thus, the results of the present study suggest that although Kp-10 treatment was not able to protect against placental hypoxia caused by maternal hypothyroidism, it was effective in protecting against the occurrence of placental oxidative damage.

Daily treatment with Kp-10 also increased the gene and/or protein expression of SOD1, catalase, and GPx1 in the placenta of hypothyroid rats. This suggests an antioxidant function of kisspeptin at the maternal-fetal interface. These results are in line with those of previous studies conducted on the ovary (43), liver (44), and testicle (45), that demonstrated increased antioxidant enzymes after kisspeptin treatment. Thus, together, these data suggest that in the condition of placental oxidative stress caused by hypothyroidism, treatment with Kp-10 can improve placental antioxidant defenses, inhibit oxidative damage, and, consequently, improve fetus and placenta development.

Furthermore, hypothyroidism increased immunolabeling in the junctional zone of endoplasmic reticulum stress mediators, GRP78, and CHOP, another pathophysiological process involved in placental dysfunction in preeclamptic women (92–94) and that has been recently demonstrated in the placenta of hypothyroid rats (40). Interestingly, although treatment with Kp-10 was not able to reverse the expression of these mediators in the junctional zone of hypothyroid rats, including with increased placental gene expression of *Grp78* and *Chop* when compared to the hypothyroid group, Kp-10 treatment reduced GRP78 and CHOP immunolabeling in the labyrinth zone of hypothyroid animals. This suggests attenuation of reticular stress in this placental layer after Kp-10 treatment. Furthermore, a recent study demonstrated *in vitro* in hypothalamic GT1-7 cells, that silencing of *Kiss1* or inhibition of *Kiss1r* with the antagonist Kp234 resulted in endoplasmic reticulum stress, while treatment with Kp-10 blocked its activation (46). Since maternal hypothyroidism in rats reduces placental and decidual expression of the Kiss/Kiss1r system (31), this may also be one of the reasons for the activation of reticular stress in the placenta of these animals (40).

The findings of this study demonstrated that daily treatment with Kp-10 in hypothyroid pregnant rats was able to improve fetus and placenta development and the plasma free T3 and T4 levels. This improvement was associated with not only increased expression of placental growth factors and antioxidant enzymes, but also with blockage of oxidative damage and positive modulation of reticular stress mediators, IL-10, and VEGF in the labyrinth zone. This is the first study to use kisspeptin as a therapeutic tool in a gestational disease.

## Data availability statement

The raw data supporting the conclusions of this article will be made available by the authors, without undue reservation.

## Ethics statement

The animal study was reviewed and approved by Ethics Committee on the Use of Animals of Santa Cruz State University (UESC) (Protocol 036/16).

## Author contributions

BS and JS conceived and designed the study. BS, JA, EB, and LS conducted sample collection. BS and EB conducted the evaluation of maternal and fetus data. BS conducted histopathological preparation and analysis. BS, JA, LS, ES, LM, IM, and JS performed immunohistochemistry analysis. BS, JA, LS, and JS performed qRT-PCR. BS and JS analyzed data. JS, ML, RES, and RS provided reagents. BS and JS wrote the paper. All authors read and approved the manuscript. All authors contributed to the article and approved the submitted version.

## Funding

This work was supported by Conselho Nacional de Desenvolvimento Cientifico e Tecnologico (CNPq), Universidade Estadual de Santa Cruz (UESC) and Coordenaçao de Aperfeiçoamento de Pessoal de Nivel Superior (Capes).

## Acknowledgments

The authors thank Ivo Arouca (*Universidade Estadual de Santa Cruz, Ilheus, Brazil*) by the technical support provided.

## Conflict of interest

The authors declare that the research was conducted in the absence of any commercial or financial relationships that could be construed as a potential conflict of interest.

## Publisher’s note

All claims expressed in this article are solely those of the authors and do not necessarily represent those of their affiliated organizations, or those of the publisher, the editors and the reviewers. Any product that may be evaluated in this article, or claim that may be made by its manufacturer, is not guaranteed or endorsed by the publisher.

## Supplementary material

Supplementary Figure 1 Immunohistochemistry. **(A–B)** Representative images of the junctional zone **(A)** and the labyrinth zone **(B)** in the negative control. **(C)** Antibodies used with their respective dilutions and incubation and revelation times.
