# Supplementary figures and images for: Kisspeptin treatment improves fetal-placental development and blocks placental oxidative damage caused by maternal hypothyroidism in an experimental rat model
Source: Front Endocrinol (Lausanne). 2022 Jul 28;13:908240. doi: 10.3389/fendo.2022.908240 (PMC9365946; doi:10.3389/fendo.2022.908240)

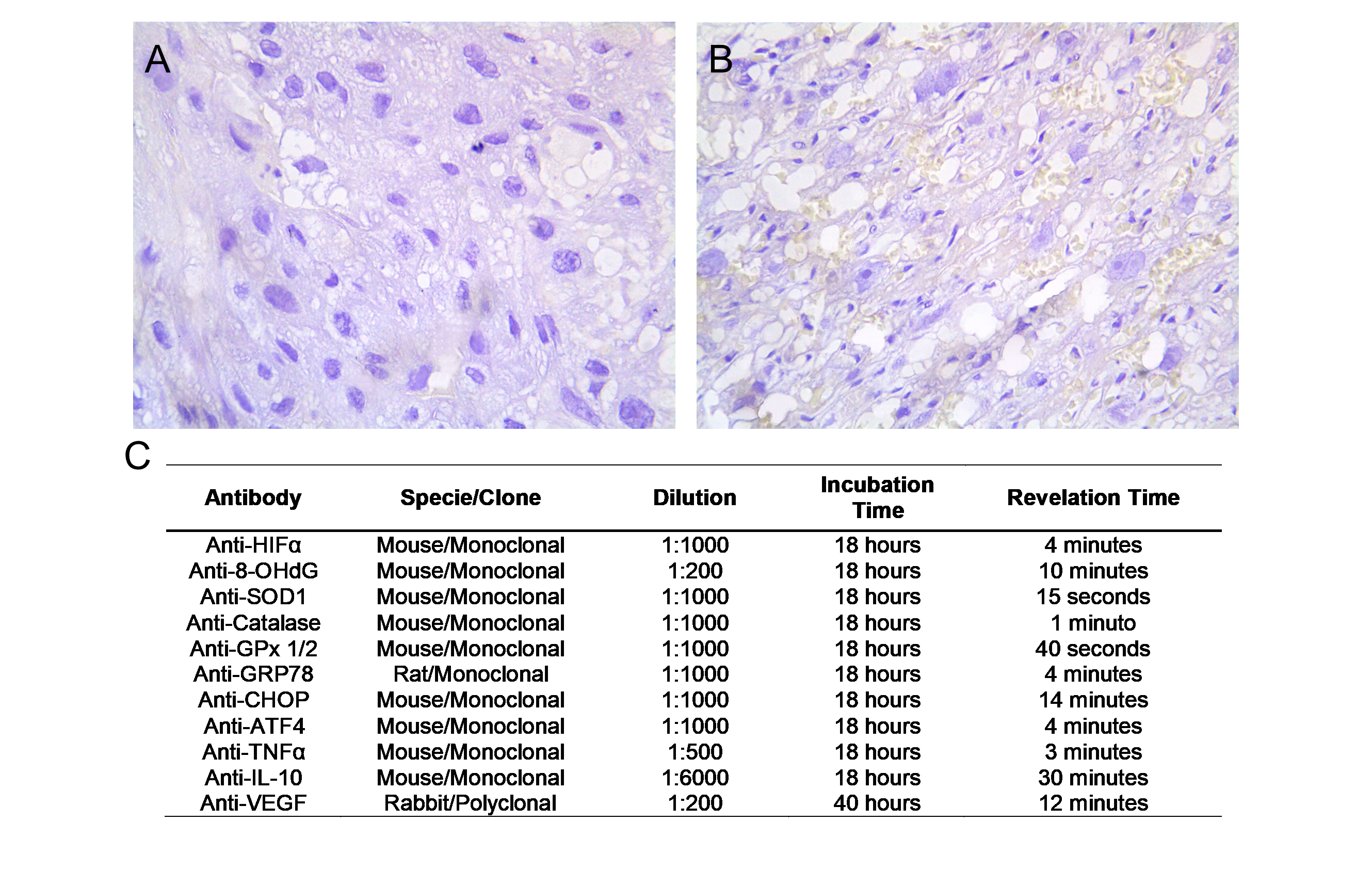

Supplement: Supplementary Figure 1 — Immunohistochemistry. (A–B) Representative images of the junctional zone (A) and the labyrinth zone (B) in the negative control. (C) Antibodies used with their respective dilutions and incubation and revelation times. [file Image_1.tif]
